# Contribution of muscle satellite cells to sarcopenia

**DOI:** 10.3389/fphys.2022.892749

**Published:** 2022-08-12

**Authors:** Fengjiao Huo, Qing Liu, Hailiang Liu

**Affiliations:** ^1^ Institute for Regenerative Medicine, Shanghai East Hospital, Tongji University School of Medicine, Shanghai, China; ^2^ Key Laboratory of Xinjiang Phytomedicine Resource and Utilization of Ministry of Education, College of Life Sciences, Shihezi University, Shihezi, China

**Keywords:** aging, sarcopenia, muscle satellite cell, regeneration, rejuvenation

## Abstract

Sarcopenia, a disorder characterized by age-related muscle loss and reduced muscle strength, is associated with decreased individual independence and quality of life, as well as a high risk of death. Skeletal muscle houses a normally mitotically quiescent population of adult stem cells called muscle satellite cells (MuSCs) that are responsible for muscle maintenance, growth, repair, and regeneration throughout the life cycle. Patients with sarcopenia are often exhibit dysregulation of MuSCs homeostasis. In this review, we focus on the etiology, assessment, and treatment of sarcopenia. We also discuss phenotypic and regulatory mechanisms of MuSC quiescence, activation, and aging states, as well as the controversy between MuSC depletion and sarcopenia. Finally, we give a multi-dimensional treatment strategy for sarcopenia based on improving MuSC function.

## 1 Introduction

The international generic diagnosis of sarcopenia encompasses aspects such as reduced muscle mass and reduced muscle strength. Prevention and intervention of sarcopenia is essential to human health, as even healthy individuals lose muscle mass and function as they age. As a syndrome, sarcopenia is caused by various factors, such as physiological aging, malnutrition, lack of exercise, disease, and inflammation. The pathogenesis of sarcopenia is complex, with hormonal disorders, reactive oxygen species (ROS) accumulation, pro-inflammatory cytokines, defective autophagy, and dysfunction of muscle satellite cells (MuSCs) adding to the complexity of its treatment. Existing therapeutic strategies for sarcopenia include exercise, dietary supplementation, and hormonal interventions that partially rescue the adverse consequences of sarcopenia by inducing autophagy, enhancing energy metabolism, suppressing inflammation, and increasing protein synthesis. MuSCs, located in a niche between the plasma membrane and basement membrane of muscle fibers, represent a heterogeneous population that is usually in a quiescent state. However, when muscle fiber injury or myotonic disease occurs, quiescent MuSCs are activated. A subset of activated MuSCs differentiates and fuses to regenerate muscle for post-injury repair, whereas another subset replenishes the quiescent satellite cell pool by inhibiting myogenic decisions. Whether it is maintenance of the quiescent state or regulation of the activated state, MuSCs are regulated by signals in the niche and circulation. With aging, MuSCs are extensively affected by environmental signals, leading to a decrease in their number and degradation of their function that affects muscle regeneration and worsens sarcopenia. As the correlation between MuSCs and muscle hypertrophy has been addressed, MuSC-based interventions for sarcopenia have been widely studied. We believe that the best interventions to protect the functionally intact MuSC pool during aging are through rescue of the cell cycle, signaling pathways, autophagy, DNA damage and epigenetics of aging MuSCs. In addition, modulation of neighboring cell signaling and substrates in the ecological niche; inhibition or supplementation of soluble small molecules such as cytokines, growth factors, and hormones in the circulation; as well as interventions that reverse the overall level of aging in the organism are likely useful measures.

## 2 Sarcopenia

### 2.1 Definition and diagnosis of sarcopenia

Sarcopenia (Rosenberg, 1997) was originally coined by Evans and Campbell as a combination of two Greek words sarx (meat) and penia (loss). In the 1980s, Irwin Rosenberg defined sarcopenia as age-related muscle mass loss. In 2010, the European Working Group on Sarcopenia in Older People (EWGSOP) defined low muscle function (characterized by low muscle strength) and reduced muscle mass as two criteria for the clinical diagnosis of sarcopenia (Cruz-Jentoft et al., 2010). In 2016, sarcopenia was recognized as an independent disease (Cruz-Jentoft et al., 2019) and in 2018, EWGSOP updated EWGSOP2 to include low muscle strength as the primary index for diagnosis of sarcopenia, and define suspected sarcopenia as the presence of decreased muscle strength. Sarcopenia is characterized by decreased muscle strength and muscle mass, whereas severe sarcopenia is characterized by decreased muscle strength, muscle mass, and physical function (Cruz-Jentoft et al., 2019). In 2020, the Asian Working Group of Sarcopenia (AWGS) updated its Expert Consensus on the Diagnosis and Treatment of Sarcopenia Asia (AWGS2019), which defines sarcopenia as low muscle mass, low muscle strength, and/or low physical function. Currently, there is no internationally accepted diagnostic standard for the definition of sarcopenia.

Diagnosis of sarcopenia is based on the definition of sarcopenia, which requires a comprehensive assessment of the patient’s muscle mass, muscle function (strength), and body function. According to the stepwise diagnostic approach and defined cutoff points proposed by EWGSOP2 (Cruz-Jentoft et al., 2019), sarcopenia is usually evaluated from grip strength, which is easy to measure and highly predictive of adverse outcomes, to detect muscle strength. According to EWGSOP2, the grip strength of men with sarcopenia is less than 27 kg, and that of women is less than 16 kg (Cruz-Jentoft et al., 2019). The second step in diagnosis of sarcopenia is measurement of muscle mass. AWGS2019 recommends using DXA or DSM-BIA combined with the height-corrected appendicular skeletal muscle mass (ASM) index (ASM/height^2^) to evaluate muscle mass. According to EWGSOP2, this ratio is less than 7 in men and 5.5 in women with sarcopenia (Cruz-Jentoft and Sayer, 2019). The concept of body function includes muscle function as well as central and peripheral nerve function, which are used to evaluate the cooperation of individual tissues and organs. The normal walking pace of patients is often used in clinical evaluation of physical function because it can predict the occurrence of adverse health events related to sarcopenia, such as disability, cognitive impairment, weakness, and fall susceptibility. According to EWGSOP2, the walking pace of men and women with sarcopenia is less than 0.8 m/s (Cruz-Jentoft et al., 2019).

### 2.2 Causes of sarcopenia

**FIGURE 1:**
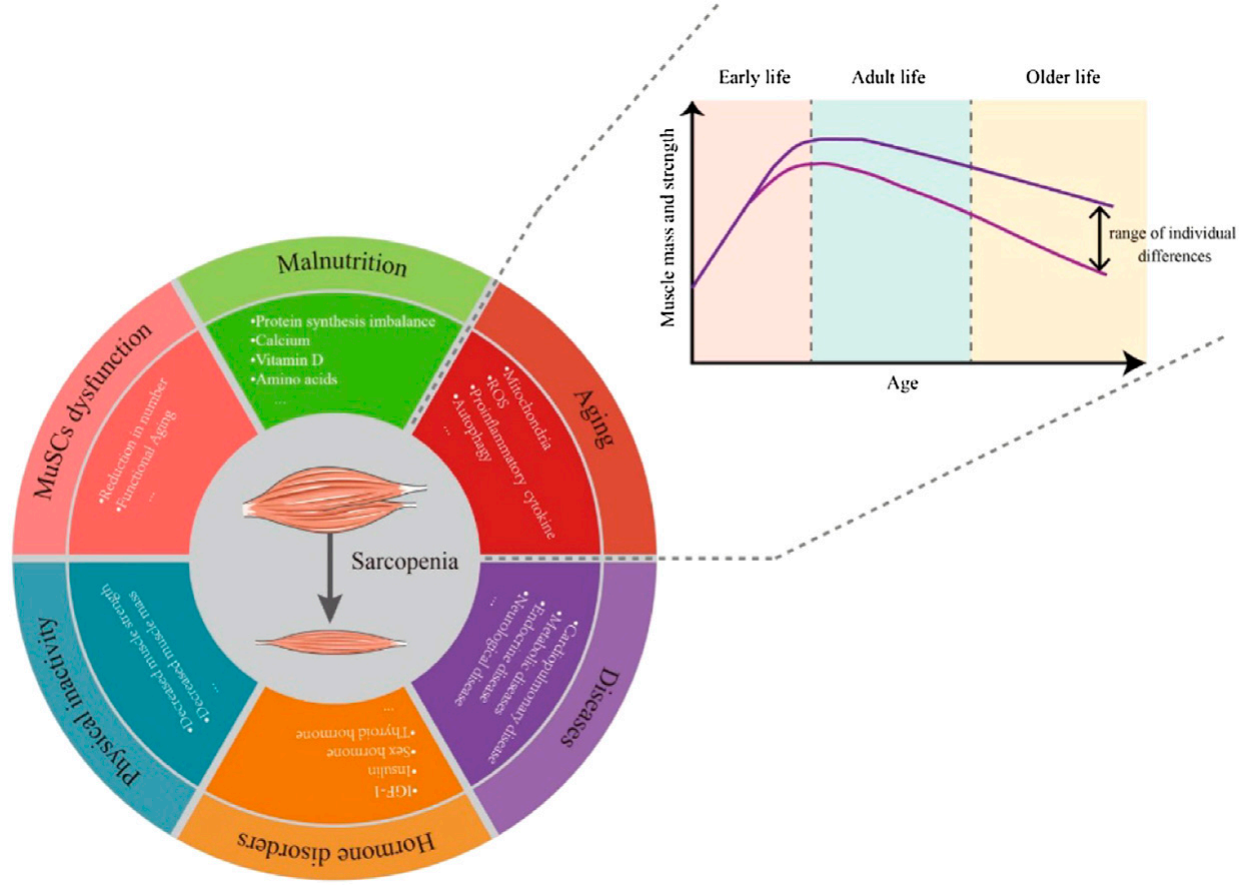
Over a lifetime, the body’s muscle mass and strength peaks in adulthood and then gradually declines with aging. The rate of decline varies between people with different lifestyles. The causes of sarcopenia include but are not limited to aging, malnutrition, MuSC dysfunction, physical inactivity, hormone disorders and diseases.

Like other syndromes, the causes of sarcopenia syndrome are varied. Even healthy individuals can gradually lose muscle mass and function with aging. During early growth and development, muscle weight gradually increases to a peak and is generally maintained subsequently. Each person loses about half a pound of muscle a year between the ages of 30 and 60 (Figure 1). But this loss of muscle mass is accompanied by a doubling of fat gain (Forbes, 1999; Barazzoni et al., 2018), so it is usually not noticeable (Gallagher et al., 2000). Changes in amounts of muscle and fat can also lead to changes in muscle composition, with fat infiltration into muscle to form “marbling” (Visser et al., 2002), which can also reduce muscle mass and performance.

Multiple factors, not just aging, can contribute to the onset and progression sarcopenia, including poor nutrition, lack of exercise (Glover et al., 2008; Tanner et al., 2015), disease, inflammation, and iatrogenic factors (Cruz-Jentoft et al., 2019). Protein intake is closely related to skeletal muscle health (Cruz-Jentoft et al., 2010; Cruz-Jentoft et al., 2014; Lorenzo-Lopez et al., 2017). In sarcopenia, muscle protein synthesis and muscle protein breakdown (MPB) are unbalanced (Paddon-Jones et al., 2008; Breen and Phillips, 2011), and the response of older people to muscle myofibrillar protein synthesis supplied by protein/amino acid is also weakened (Cuthbertson et al., 2004; Katsanos et al., 2005; Moore et al., 2015; Brook et al., 2016). Long-term bed rest or an otherwise sedentary or low-exercise lifestyle negatively impact the volume, structure, chemical composition, and muscle nerve excitability of muscle fibers (Zampieri et al., 2015), which accelerates the process of sarcopenia. Chronic heart and lung diseases (such as chronic heart failure, chronic obstructive pulmonary disease) (Buchman et al., 2009; Bouillon et al., 2013; Elbaz et al., 2014; Navarro-Cruz et al., 2019), diabetes (Sinclair et al., 2017), metabolic diseases (Cleasby et al., 2016), endocrine diseases (such as androgen deficiency or deprivation), cancer, liver, and kidney diseases, bone and joint diseases, and even nervous system disorders can aggravate sarcopenia by restricting the absorption of nutrients and increasing inflammation.

Mechanisms of sarcopenia are complex and varied. Reduction of insulin-like growth factor-1 (IGF-1), a major anabolic signal of muscle protein synthesis, is associated with skeletal muscle mass (Ari et al., 2004; Bian et al., 2020), lower grip strength (van Nieuwpoort et al., 2018), and reduced physical performance. Insulin is also an anabolic hormone acting on myocyte (Bouchi et al., 2017; Tokarz et al., 2018). The sensitivity of myocyte to insulin decreases during aging, resulting in a decrease of muscle mass. In addition, elevation of glucocorticoids under stress, cachexia, and other conditions can increase MPB (Hoppeler, 2016). Decreased numbers of mitochondria and mitochondrial dysfunction occurring with aging can lead to muscle reduction and functional decline (Chabi et al., 2008; Wawrzyniak et al., 2016; Picca et al., 2018). Simultaneously, accumulated ROS leads to production of faulty proteins and overall dysfunction of mitochondria (Chabi et al., 2008; Chocron et al., 2019), which reduce muscle function. One of characteristic of aging is the increase of circulating proinflammatory cytokines [such as interleukin 1 (IL-1), IL-6, tumor necrosis factor (TNF-α), and C-reactive protein] that can promote MPB (Argiles, 2017; Dalle et al., 2017) and downregulate IGF-1 (Piotrowicz and Gasowski, 2020), thereby affecting muscle strength and activity. In addition, decreases of autophagy associated with aging can lead to accumulation of damaged cell components and misfolded proteins (Mizushima et al., 2008), as well as decreased numbers and function of MuSCs (Garcia-Prat et al., 2016), which affect the function and regeneration of myocytes (Garcia-Prat et al., 2016; Jiao and Demontis, 2017). The negative effects of MuSC quiescence and impaired self-renewal capacity on sarcopenia will be discussed in the next section.

### 2.3 Treatment of sarcopenia

Several approaches, including exercise, dietary supplements, and hormonal interventions are presently the main interventions for managing sarcopenia. Exercise reduces age-related oxidative damage and chronic inflammation, increases autophagy, improves mitochondrial function, and enhances IGF-1 signaling and insulin sensitivity. Resistance exercise (RE) is generally considered to alleviate sarcopenia by increasing skeletal muscle mass and strength (Cruz-Jentoft and Landi, 2014; Landi et al., 2014; Karlsen et al., 2020). Mechanistic studies found that RE can reduce age-induced intracellular lipid (IMCL) accumulation and enhance expression of glucose homeostasis regulatory components (e.g., glucose transporter type 4, glucose-6-phosphate dehydrogenase, hexokinase 2, and glycogen synthase 1) to resist sarcopenia (Ribeiro et al., 2017). RE can also induce autophagy, which is closely related to sarcopenia (Masiero et al., 2009; Sandri, 2010), by regulating Akt/mechanistic target of rapamycin (mTOR) and Akt/FOXO3a signaling pathways, as well as AMPK-mediated mitochondrial quality control (Zeng et al., 2020). Most exercise interventions currently used to treat sarcopenia involve a combination of aerobic, balance, and resistance exercises, which are more effective than RE alone (Moore et al., 2020). However, even in elderly individuals who are physically active, the loss of muscle weight and increase in body fat percentage are still significant (Hawkins et al., 2001), so exercise is not a radical cure.

Nutritional supplementation combined with exercise intervention is a very common treatment for sarcopenia (Abizanda et al., 2015; Naclerio and Larumbe-Zabala, 2016; McKendry et al., 2020). In young individuals, RE makes the body more sensitive to protein supplemented in the diet (Symons et al., 2009). During this time, a combined approach of RE with intake of more protein can be directly used for skeletal muscle synthesis. However, owing to problems related to anabolic resistance (Burd et al., 2013), older people need to eat more protein to maintain muscle mass and function. Protein intake of 1.0–1.3 g/kg/day reduced muscle mass loss in older people by 40% (Houston et al., 2008; Valenzuela et al., 2013) compared with the previously recommended (Manore and Meacham, 2003; Wolfe et al., 2017) intake of 0.66–0.80 g/kg/day. Balanced meal frequency and total protein intake at each meal are more beneficial to MSP in older people (Loenneke et al., 2016; Smeuninx et al., 2020). Notably, animal-derived proteins are more widely recommended than plant-based proteins for sarcopenia owing to their comprehensive amino acids (Gorissen et al., 2018; Churchward-Venne, 2019), high digestibility, and low anti-nutritional factors (Gilani et al., 2012). In particular, branched-chain amino acids, including leucine, valine, and isoleucine, can increase muscle mass by directly promoting protein synthesis through activation of the mTOR signaling pathway (Katsanos et al., 2006; Le Couteur et al., 2020). In addition, the leucine metabolite β-hydroxy-β-methylbutyrate has a positive effect on protein synthesis and mitochondrial dynamics in muscle (Sanz-Paris et al., 2018; Standley et al., 2020), and its oral administration of together with other protein supplements is beneficial for maintaining muscle mass in older people (Sanz-Paris et al., 2018). Supplementation with omega-3 polyunsaturated fatty acids, polyphenols, and vitamin D can also suppress inflammation and improve metabolism, thereby supporting skeletal muscle function (Domingues-Faria et al., 2016; Dupont et al., 2019; Du et al., 2020).

Hormonal modulation, a classic strategy to alleviate sarcopenia (Kwak and Kwon, 2019), offers new options for elderly individuals suffering from exercise intolerance. One of the key options is to promote myogenic cells and protein synthesis by activating androgen receptor (AR) expression in satellite cells and myoblasts. Testosterone is age-dependently reduced as a ligand for ARs. Supplementation with testosterone promotes satellite cell entry into the cell cycle, thereby increasing the number of satellite cells (Hikim et al., 2002). Moreover, testosterone increases the response of fusion-impaired myoblasts, enhances AR levels, and increases the abundance of AKT expression (Hughes et al., 2016; Pal et al., 2019). In addition, testosterone improves the efficiency of amino acid utilization after proteolysis and promotes muscle synthesis (Ferrando et al., 1998). Experiments have proven that testosterone can increase muscle protein synthesis in frail and elderly patients with heart failure, thereby improving muscle strength and increase walking distance. However, there are risks associated with testosterone therapy, including sleep apnea, thrombotic complications, and increased risk of prostate cancer. Accordingly, the potential risks of testosterone use in older people population may outweigh its benefits, so it is not widely used. Selective androgen receptor modulators (SARMs), a new class of AR ligands, have the same effect as androgens with higher selectivity for bone and muscle tissues with fewer side effects. Embosarm, for example, successfully increased muscle mass and body functions in patients without cancer and patients with advanced lung, colorectal, breast cancer, or lymphoma. The Embosarm Phase II study in 120 healthy older adults showed both lean body mass gain and dose-dependent improvement in stair climbing (Dalton et al., 2011). However, Embosarm has not been approved by the United States Food and Drug Administration owing to hepatotoxicity and unpredictable post-administration prognosis.

Melatonin, an effective substitute for testosterone, was shown to prevent muscle atrophy induced by the IGF-1 axis in rats. In addition, melatonin can reduce adipose formation in obese mice by regulating autocrine and paracrine responses in muscle and adipose tissue (Jin et al., 2021).

## 3 Muscle satellite cells

As the tissue with the largest body mass, skeletal muscle is composed of myofibers fused by large numbers of myogenic progenitor cells, which affect body movement, respiration, and metabolism. Skeletal muscle can maintain its size and function through regeneration after injury thanks to resident MuSCs, also known as “satellite cells”, which were discovered in 1961 by Mauro in vertebrate muscle using electron microscopy (Mauro, 1961). MuSCs are located in a unique anatomical position between the myofiber plasma membrane and basal lamina (Mauro, 1961), and normally reside in a quiescent state. When muscle fiber injury or muscular dystrophy occurs, changes in niche signaling cause quiescent satellite cells (QSCs) to become activated satellite cells (ASCs) (Wosczyna and Rando, 2018). A subset of ASCs subsequently differentiates and fuse to regenerate muscle for post-injury repair, whereas another subset replenishes the QSC pool by inhibiting myogenic determination (Evano et al., 2020).

Normally, MuSCs stay within their stable, although complex, niche. Single-cell RNA sequencing revealed the diversity of cell types in the MuSC niche, including resident macrophages, T cells, B cells, neutrophils, endothelial cells, fibroadipogenic progenitor cells (FAPs), Schwann cells, tenocytes, and smooth muscle-mesenchymal cells (Mashinchian et al., 2018). MuSCs and myofibers can interact directly [e.g., through muscle cadherins (M-CADH) and the Notch signaling pathway] (Goel et al., 2017; Baghdadi et al., 2018) or indirectly by expressing related receptors in response to cytokines produced by other cells in the niche (e.g., hepatocyte growth factor receptor and epidermal growth factor receptor) (Tatsumi et al., 1998; Bentzinger et al., 2010). In addition, MuSCs can also interact with extracellular matrix (ECM) components, such as the interaction between laminin-integrin and calcitonin receptor (CALCR) expressed on the surface of MuSCs (Baghdadi et al., 2018).

### 3.1 MuSC quiescence and activation

#### 3.1.1 Characteristics of MuSC quiescence

Cell surface markers of QSCs are distributed unevenly on the cell surface, including CD34, c-Met, syndecans 3 and 4, integrins α7 and β1, neural and vascular cell adhesion molecules, neural and muscle cadherins (N-CADH and M-CADH), C-X-C motif chemokine receptor 4, CALCR (Zhang et al., 2019), and calveolin 1 (Feige et al., 2018; Garcia-Prat et al., 2020), which jointly control QSC quiescence by integrating signals from the external niche and intercellular interactions.

**FIGURE 2:**
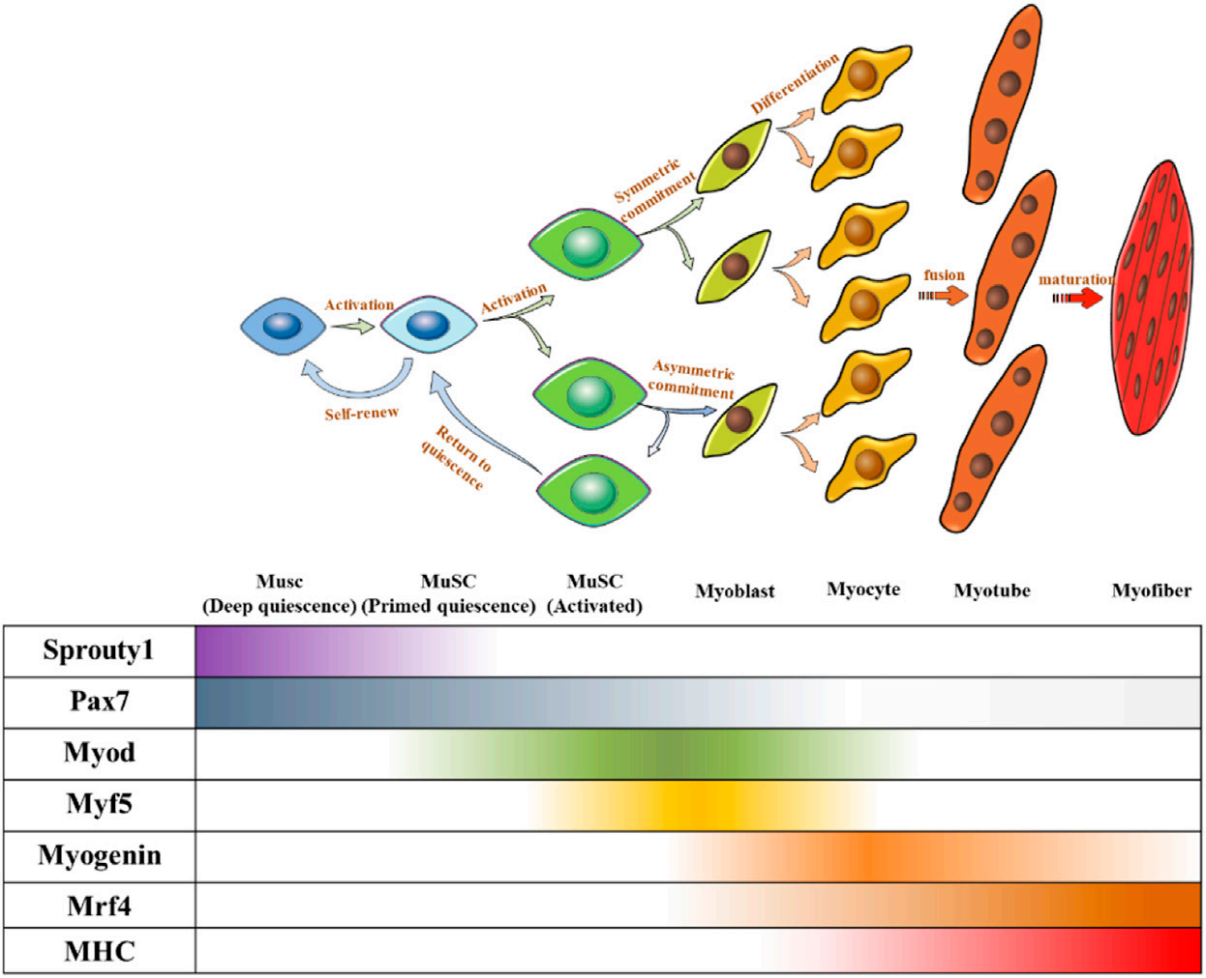
Fate regulations of MuSC towards muscle differentiation and self-renewal. Quiescent MUSC express Pax7, and symmetric division upon MuSC activation can produce two identical MuSCs to maintain a stable MuSC pool, and asymmetric division can produce one MuSC, one myogenic committed cell. Myogenic committed cells express MRFs step-by-step to differentiate into myocytes. Myocytes stop proliferating and fuse to form multinucleated myotubes. Myotubes further mature and bundle together to form myofibers.

Under normal circumstances, MuSCs reside in a quiescent state in adults (Cheung and Rando, 2013). The quiescence and activation of MuSCs are crucial for maintaining the stem cell pool and muscle regeneration (Relaix and Zammit, 2012), which require precise regulation. Several transcription factors have been identified as key regulators and markers of MuSCs and their progression toward myogenic lineages. Paired-box transcription factor (Pax7) is a canonical marker of MuSCs because of its continuously high expression, in which some subgroups co-express Pax3 (Buckingham and Relaix, 2015). Hierarchical expression pattern of myogenic regulatory factors (MRFs) including Myf5, MyoD, MRF4, and myogenin determine the sequential control of myogenic development (Relaix and Zammit, 2012). In general, QSCs express Pax7 without expressing MRFs. When QSCs were activated to proliferate and differentiate into myoblasts, Pax7 expression decreased and MRFs began to be expressed. Myoblasts expressed MyoD and MYF5, whereas Pax7 was lowly expressed. Subsequently, myoblasts differentiated into myocytes expressing both myogenin and MRF4 (Almada and Wagers, 2016), before further fusing to form multinucleated myotubes. Finally, myotubes combine to form muscle fibers (Figure 2).

### 3.2 Regulations of MuSC quiescence

The quiescence of MuSCs is jointly regulated by extracellular cues and intracellular molecules. Abnormal activation of QSCs leads to the accumulation of aging-related damage and depletion of the stem cell pool (Chakkalakal et al., 2012; Kimmel et al., 2020). Therefore, ensuring accurate expression of these regulatory factors is very important for muscle regeneration.

#### 3.2.1 Extracellular cues on MuSC quiescence

Owing to the anatomical location of QSCs, muscle fibers and ECM play important roles in maintenance of their quiescent state. N-CADH and M-CADH expressed on the surface of QSCs are in direct contact with muscle fibers, and their genetic removal leads to QSC activation. The ligand Delta-like 1 located on muscle fibers can bind to Notch1–3 receptors expressed by QSCs, causing translocation of the Notch intracellular domain to the nucleus, whereby it regulates expression of quiescence-related genes (e.g., Hes/Hey family genes) (Fujimaki et al., 2018; Yartseva et al., 2020). The role of Notch signaling in maintenance of MuSC quiescence is well characterize (Bjornson et al., 2012). Interference of Notch signaling can damage MuSC self-renewal, thereby causing spontaneous differentiation and depletion of the stem cell pool. Activation of KLF7, a downstream target of Notch important for maintaining MuSC quiescence, induces upregulation of the cyclin-dependent kinase (CDK) inhibitor P21 ^CIP1^, which inhibits MuSC entry into the cell cycle (Wang et al., 2016). FOXO, a transcription factor highly expressed in QSCs, can further consolidate Notch signaling. In MuSCs with a disturbed FOXO axis, constitutive Notch activation can re-establish MuSC quiescence and prevent their depletion (Gopinath et al., 2014). Moreover, Notch signaling was found to induce miR-708 transcription, which inhibits MuSC migration by anchoring them within the niche and promoting maintenance of a quiescence state (Baghdadi et al., 2018). The MuSC surface marker CALCR is critical for maintaining MuSC quiescence and anchoring it in its niche, and the progressive decrease of CALCR with aging may contribute to MuSC loss. Given that the intrinsic ligand of CALCR, collagen V (COLV) (Ikemoto-Uezumi et al., 2019), and its upstream regulator Notch signaling (Baghdadi et al., 2018) are unaffected by age, targeting CALCR or its downstream signaling may be a therapeutic strategy to rescue age-related loss of MuSCs.

Paracrine regulation within the MuSC niche cannot be ignored. Serial transplantation assays showed that oncostatin M, a member of the IL-6 cytokine family secreted by muscle fibers, could protect the quiescent state of MuSCs and sustains their stemness (Sampath et al., 2018). Wnt4, another cytokine secreted by muscle fibers, can delay MuSC entry into the cell cycle and activate Rho-GTPase acting on the cytoskeleton, causing mechanical sclerosis of MuSCs (Eliazer et al., 2019). Wnt4 also inhibits transcription of Yap, a key cellular mechanotransducer implicated in the mechanical process of cell regeneration (Sun et al., 2017), thereby maintaining the quiescent state of MuSCs.

Other components in the niche, such as the key basal lamina component laminin, provide binding sites for integrins α7 and β1 expressed on the surface of MuSCs to anchor MuSCs to the ECM and provide physical support for their quiescence. Skeletal muscle is rich in blood vessels. Interactions between vascular endothelial cells and MuSC participate in the maintenance MuSC quiescence and MuSC-mediated muscle regeneration. VEGF secreted by satellite cells recruits vascular endothelial cells. Notch ligand Delta-like 4 expressed on endothelial cells activates Notch signaling of MuSCs, which maintains their quiescence and inhibits their myogenic progression (Verma et al., 2018). FAPs have the ability to differentiate into fibroblasts, adipocytes, osteoblasts, and chondrocytes under specific circumstances. FAP-depleted mice exhibited symptoms of MuSC loss and muscle atrophy (Wosczyna et al., 2019), although whether this occurs through maintenance of MuSC quiescence remains to be studied. There are few immune cells in homeostatic skeletal muscle, but their number rapidly increases after muscle injury, which will affect MuSC activation. For example, *in situ* phenotypic transformation from pro-inflammatory macrophages to antigen macrophages can stimulate differentiation of MuSCs (Perdiguero et al., 2011). Moreover, a macrophage subgroup identified in zebrafish secreted nicotinamide phosphoribosyltransferase, which bound to CCR5 receptors of MuSCs to stimulate their mitosis, thus promoting regeneration after injury (Ratnayake et al., 2021).

#### 3.2.2 Intracellular molecules on MuSC quiescence

To maintain QSCs in stasis at the G0 phase of the cell cycle, transcription factors driving cell cycle progression and myogenic differentiation must be minimally expressed. In contrast, PAX7 and stemness genes related to cell regeneration and differentiation were highly expressed (Pietrosemoli et al., 2017). The cell cycle of MuSCs is regulated by cyclins (cycA, cycB, cycC, cycD, and cycE) and CDK, whose expression is regulated by the E2F family. Activity of E2F family members is inhibited by CDK inhibitors of the CIP/KIP and INK4A families, thus limiting the mitotic process of QSCs. For example, activation of p21^CIP1^, downstream of the Notch signaling pathway, inhibits QSC entry into the cell cycle (Wang et al., 2016). P57 translocates from the cytoplasm to the nucleus, causing myogenic myoblasts to exit the cell cycle (Wang et al., 2016). p27 ^KIP1^-Null mice exhibited reduced MuSC reserves and MuSCs that could not be restored to quiescence after two rounds of serial injuries, resulting in stem cell pool depletion. Examination of involved signaling pathways revealed upregulation of TNF/nuclear factor κB (NF-κB), IL-6-Janus kinase (JAK)-STAT3, AMP-activated protein kinase (AMPK), and Notch signaling pathways. Moreover, genes associated with cell cycle progression (e.g., G2/M cell cycle checkpoint-related genes) were downregulated in QSCs.

Post-transcriptional regulation is also important for maintaining MuSC quiescence. Previously, we mentioned that Mir-708 anchors MuSCs in the niche to maintain their quiescence (Baghdadi et al., 2018). Indeed, multiple miRNAs were found to be involved in QSC maintenance. Mir-489 targets Dek mRNA to inhibit maturation and processing of myogenic transcripts (Cheung et al., 2012), whereas Mir-31 binds Myf5 mRNA to ribonucleoprotein granules to prevent their translation (Crist et al., 2012). Matrix metalloproteinase 9, which degrades skeletal muscle matrix, prevents MuSCs from quiescence and regeneration. AUF1 targets the 3′ AU-rich elements of matrix metalloproteinase 9 mRNA to counteracts its adverse effects (Chenette et al., 2016). Similarly, Staufen1 prevents translation of MyoD by interacting with the MyoD 3′ untranslated region (de Morree et al., 2017). Simultaneously, the MyoD mRNA 3′ untranslated region can bind to tristetraprolin, which promotes its decay (Hausburg et al., 2015).

Autophagy refers to the process of self-degradation of organelles, cytoplasmic parts, and misfolded proteins in lysosomes, which is essential for maintaining MuSCs in a quiescent state. Impaired autophagy activity in elderly MuSCs accelerates stem cell depletion (Garcia-Prat et al., 2016; Wang et al., 2016). Genetic inhibition of autophagy-related genes in healthy young mice results in rapid MuSC senescence, resulting in a decreased MuSC numbers and function, thus affecting muscle regeneration (Garcia-Prat et al., 2016).

The importance of epigenetic modifications on gene expression is also reflected in the control of MuSC quiescence (Liu et al., 2013). For example, the NAD^+^-dependent deacetylase SIRT1 associates histone acetylation with MuSC fate by sensing NAD^+^ levels (Feige et al., 2008). The NAD^+^ level of QSCs is 10 times higher than that of ASCs. During quiescence, SIRT1 inhibits the expression of genes related to MuSC cell cycle entry and myogenic development by deacetylating histone H4K16 (Ryall et al., 2015). Evidence shows that MuSC-specific deletion of SIRT1 can induce abnormal expression of MyoD and cause MuSC activation (Ryall et al., 2015).

### 3.3 MuSC activation and cell fate

Just as MuSCs are regulated to remain quiescent, their activation is also directly influenced by cell cycle regulators. Perturbation of the signaling pathways and regulators that maintain MuSC quiescence activates QSCs to enter the cell cycle. As the regulatory center of MuSC activation, PI3K-AKT mediates the activation of mTOR signaling and inhibits high expression of FOXO in QSCs, which is closely related to their activation.

In 2014, Rodgers et al. reported the existence of an intermediate state (G_alert_) between quiescence and activation of MuSCs (Rodgers et al., 2014). Compared with QSCs, G_alert_ MuSCs were in a state of mobilization, with larger cell sizes and higher mitochondrial activity (Rodgers et al., 2014). Later studies confirmed that hepatocyte growth factor activator generated from tissue injury binds to C-Met on the surface of MuSCs, whereby it activates mTORC1 through PI3K to trigger the G_alert_ state of MuSCs (Rodgers et al., 2017).

MuSCs undergo two cell division patterns: symmetrical division to produce two stem cells and asymmetric division to produce one differentiated cell and one stem cell. MuSCs produced by symmetric division and the offspring MuSC produced by asymmetric division need to return to quiescence to supplement the MuSC pool. In contrast, myogenically committed cells produced by asymmetric division need to differentiate and fuse to form muscle fibers (Figure 2). Regardless of the division mode and ultimate cell fate, both processes are accompanied by withdrawal from the cell cycle.

p38α stimulates cell cycle exit by inducing expression of cell cycle inhibitors. In addition, p38α induces MuSC myogenic differentiation by activating MyoD and MEF2 transcriptional activities (Aziz et al., 2010) and promoting the action of chromatin remodeling complex on myogenic promoters (Wu et al., 2000; Forcales et al., 2012). MyoD can also induce expression of muscle-specific miRNAs (miR-206 and miR-133), downregulate Pax7, and activate the main regulator of differentiated myogenin through transcriptional (Dey et al., 2011; Dong et al., 2020) and post-transcriptional mechanisms. Finally, MyoD and myogenin work together to activate MRF4 and other muscle-derived terminal differentiation genes, causing the cell to exit the cell cycle (Bi et al., 2017).

Induction of myogenic differentiation should be avoided in the selection of a self-renewal fate during MuSC proliferation. p38-mitogen-activated kinase (MAPK) (Bernet et al., 2014), fibroblast growth factor (FGF)-extracellular-regulated kinase (ERK)/MAPK (Pawlikowski et al., 2017) and JAK-STAT3 (Pietrosemoli et al., 2017) pathways are involved in determination of the self-renewal fate. During asymmetric division and in contrast to the myogenically committed cell, the self-renewing MuSC daughter cell exhibits Notch3 receptor expression, Notch pathway activation, increased Pax7 expression, and inhibited MyoD expression, which induce the renewed MuSC to resume quiescence.

At present, how MuSCs choose a differentiation or self-renewal fate is not fully understood. *In vivo* and *in vitro* studies found that the division pattern and number of MuSCs depend on their internal counting mechanism (Evano et al., 2020), indicating the complexity of their proliferative regulation. Activation of QSCs and subsequent determination of their cell fate are precisely controlled by a variety of signals and factors.

### 3.4 Heterogeneity of MuSCs

Not all stem cells within a tissue are homogeneous, and this is true of MuSCs. Indeed, heterogeneity is considered a beneficial feature of stem cells, enabling them to improve their ability to adapt to the environment and respond to signals. The complex quiescent and active regulatory networks of MuSCs are also inseparable from their heterogeneity. Accordingly, the heterogeneity of MuSCs can be described from multiple perspectives.

First, MuSC subsets can be distinguished according to different cell surface markers. CD34 is the first candidate, according to which QSCs can be divided into two subgroups: CD34^hi^ and CD 34^low^. The former exhibits more stem-like properties, but represents no more than 15% of total cells, whereas the latter is more committed to myogenic differentiation (Garcia-Prat et al., 2020). In addition, expression of CD9 alone or in combination with CD104 can be used to distinguish between quiescent and activated myogenic progenitor cell populations (Porpiglia et al., 2017)—the CD9^+^/CD104^−^ subgroup of MuSCs is more likely to self-renew (Porpiglia et al., 2017).

MuSC activation and subsequent myogenic differentiation or self-renewal are continuously regulated by transcription factors, which are regarded as myogenic markers. Thus, QSC heterogeneity can also be defined by the expression patterns of transcription factors. Previous studies linked the loss of Myf5 expression to the maintenance of MuSC quiescence. However, with more widespread use of lineage-tracing techniques, a subset of Pax7^+^ was identified in mice that never express Myf5. Compared with Myf5-expressing subpopulations, Pax7^+^/Myf5- MuSCs performed better at reconstructing the quiescent MuSC pool after transplantation, suggesting that Pax7^+^/Myf5^−^ MuSCs retained more stemness (Kuang et al., 2007). In addition, Pax7^hi^ and Pax7^low^ MuSCs were identified by their relative expression of Pax7, with Pax7^hi^ MuSCs exhibiting a more quiescent state with lower metabolic activity (Rocheteau et al., 2012).

MuSC heterogeneity can also be distinguished according to differences in gene expression, which can be confirmed by single-cell sequencing (scRNA-seq). MuSC subsets encoding growth arrest-specific protein (Gas1), Notch-targeted Hes1, Pax7, and CALCR genes are more quiescent. MuSC subsets expressing ribosomal genes Myf5 and MyoD were more inclined to exhibit early activation (Dell'Orso et al., 2019).

To determine whether labeling was retained during proliferation, MuSCs were artificially labeled with a histone H_2_B pulse-chase system or the fluorescent dye PKH26. MuSCs that retained the label (H_2_B-GFP^+^) exhibited a low degree of differentiation, preference for self-renewal to maintain the stem cell pool, and high expression of cell cycle inhibitors p21 and p27. Unlabeled MuSCs (H_2_B-GFP-) retained fast division speeds and tended to undergo myogenic differentiation. Similarly, MuSCs exhibiting high retention of the fluorescent dye PKH26 exhibited higher stemness than MuSCs exhibiting low retention.

### 3.5 MuSCs in aging

Aging impairs both autonomous and niche-dependent regulation of MuSCs, eliciting changes in their ability to maintain quiescence and functional loss of self-renewal and regeneration—the main factors driving age-related muscle changes (Carlson et al., 2008; Sousa-Victor et al., 2014). External cues, such as changes in inflammatory factors and extracellular components [e.g., Wnt ligands (Brack et al., 2007), transforming growth factor β (TGF-β) (Carlson et al., 2008), and FGF2 (Chakkalakal et al., 2012)], may influence MuSC function. In addition, intrinsic factors including oxidative stress, DNA damage, altered signaling pathways, protein damage, and metabolic changes, can impair MuSC function (Sousa-Victor et al., 2014).

The mainstream view is that the number of MuSCs decreases in an age-dependent manner (Day et al., 2010; Verdijk et al., 2012; Verdijk et al., 2014). The data showed a significant decrease in the number of MuSC in type II muscle fibers of the vastus lateralis muscle in older (50–59 years) and senescent (70–86 years) populations compared with younger adults (18–49 years) (Verdijk et al., 2014). However, other studies show no significant difference in the number of MuSCs between young and aged mice (van der Meer et al., 2011). Therefore, this review is skeptical about the correlated changes between MuSCs and aging. In any case, there are various mechanisms for aging to affect the number of MuSCs, as suggested by the mainstream view. Although QSCs are in a dormant state of low metabolism, they still exhibit continuous autophagy to remove damaged proteins and organelles. With age, this protective mechanism is gradually lost (Sandri, 2010). Aging-dependent autophagy impairments or genetic damage of MuSCs will lead to the loss of protein homeostasis and increased oxidative stress, which further promotes aging, resulting in declining numbers and function of MuSCs (Revuelta and Matheu, 2017). These negative effects can be reversed by genetic and pharmacological manipulation. MuSCs isolated from elderly mice exhibited increased numbers of lesions containing the DNA damage marker γH2AX (Sinha et al., 2014), suggesting a potential influence of genomic instability on muscle aging. Owing to the non-mitogenic nature of QSCs, repair of DNA damage is achieved by non-homologous end joining, a repair mechanism different from cell cycle-dependent double-strand break repair (Ferdousi et al., 2014). However, because non-homologous end joining does not require template strands for DNA repair, its error rate is increased, thus increasing the possibility of cell mutagenesis (Burkhalter et al., 2015).

Insulin and IGF provided by aging muscle activate the PI3K-Akt signal pathway (classical pathway regulating the transformation of QSCs into ASCs), which subsequently activates expression of mTORC1 and inhibits expression of FOXO. Chronic activation of mTORC1 associated with aging or repeated stimulation of mTORC1 to maintain ASC proliferation and differentiation leads to depletion of the MuSC pool (Haller et al., 2017). Moreover, reduced inhibition of FOXO activity by Akt promotes expression of myogenic regulatory factors (e.g., MyoG), leading to the withdrawal of the MuSC from its quiescent state. Measures to inhibit mTORC1 activation are conducive to protection of the MuSC pool. FGF2, another cytokine whose production is increased in aging muscle (Bernet et al., 2014), binds FGF receptors on MuSCs to cause their continuous activation, which also causes MuSC depletion (Bernet et al., 2014). Overexpression of SPRY1 can counteract increases of FGF2 and protect the quiescence of aging MuSCs (Chakkalakal et al., 2012). Other studies evaluating signaling pathways found that Notch-dependent activation of p53 was decreased during regeneration of aging MuSCs, which led to impaired MuSC self-renewal and increased mitosis-related mortality of MuSCs (Liu et al., 2018). Restoring the activity of p53 in aging MuSCs can promote their survival and regeneration. Notably, a reduction of AMPK/p27 pathway activation was observed in aging MuSCs, which exhibited impaired autophagy and an increased propensity for apoptosis, thus consuming the MuSC pool (White et al., 2018).

During aging, the loss of MuSC stemness and their withdrawal from the quiescent state caused by abnormal activation can occur simultaneously. TGF-β promotes expression of CDK inhibitors and inhibits MuSC proliferation through the pSmad3 intracellular pathway (Carlson et al., 2008). Notch signaling can antagonize this function by removing pSmad3 from the promoter of CDK inhibitor genes. Therefore, continuous enhancement of TGF-β signaling and continuous consumption of Notch signaling in aging muscle may lead to increased expression of CDK inhibitors, which are related to defects in proliferation and differentiation (Carlson et al., 2008). Accordingly, blocking TGF-β signal transduction is beneficial to reducing aging and promoting MuSC function. The key reason for the loss of regenerative potential of MuSCs with aging is upregulated expression of the CDK inhibitor p16^INK4a^, which helps MuSCs permanently withdraw from the cell cycle, resulting in impaired self-renewal ability (Sousa-Victor et al., 2014). Factors associated with high expression of p16^INK4a^ include ROS accumulation in mitochondria, constitutive activation of p38/MAPK signaling (Cosgrove et al., 2014), and low expression of the p16^INK4a^ repressor Slug (Li et al., 2019). Silencing of p16^INK4a^ in MuSCs by gene knockdown or overexpression of Slug restores MuSC quiescence and rescues their ability to transplant and return to quiescence. During mitosis occurring with muscle regeneration, p53 can inhibit and defect the spindle assembly, causing apoptosis of MuSCs in the process of regeneration (Liu et al., 2018).

## 4 MuSCs and sarcopenia

**FIGURE 3:**
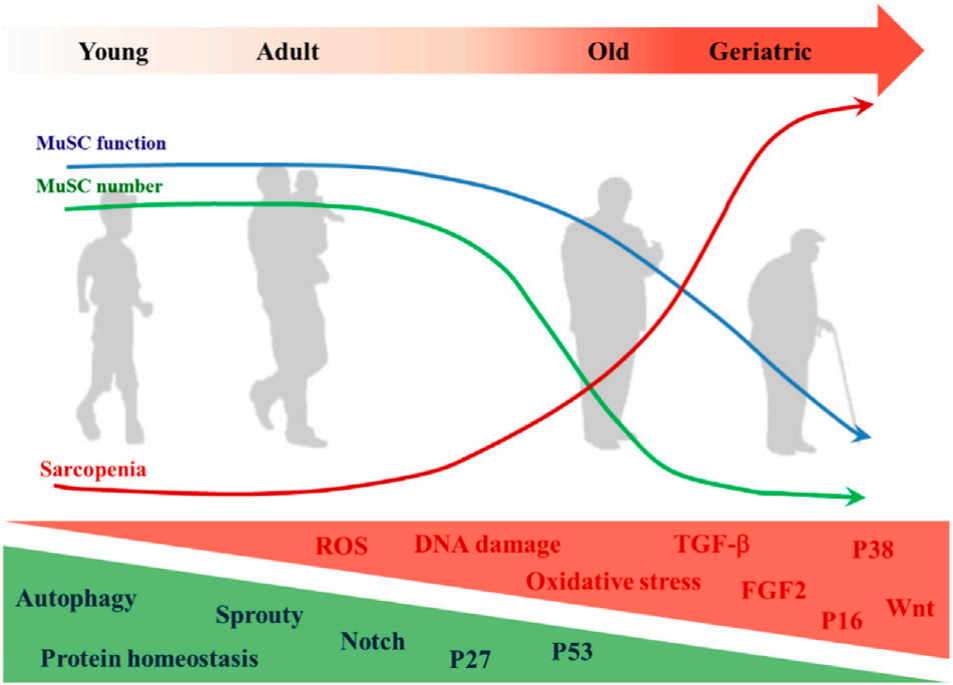
Changes of MuSC in aging. The decrease in MuSC number and functional degradation in aging is accompanied by the onset and development of sarcopenia. MuSC internal changes that increase with aging include but are not limited to: ROS, DNA damage, oxidative stress, expressions of TGF-β, FGF2, p38, p16^Ink4^, and Wnt signaling pathway. Those that decrease with aging include but are not limited to: autophagy, protein homeostasis, expressions of Sprouty, p27^Kip1^, p53, and notch signaling pathway.

There is a correlation between MuSC-dependent decline in muscle regenerative capacity and age (Sadeh, 1988), and this phenomenon is particularly evident in sarcopenia (Welle, 2002; Sousa-Victor and Munoz-Canoves, 2016). It has also been suggested that sarcopenia can be separated from MuSC dysfunction (Fry et al., 2015), as sarcopenia occurs before MuSC impairment (Sousa-Victor et al., 2014). The etiology of sarcopenia is complex and diverse (Figure 3). Although the role of MuSCs in sarcopenia is questionable, maintaining the integrity of MuSC number and function is indispensable for muscle health (Keefe et al., 2015).

### 4.1 Relationship between MuSCs and sarcopenia

The loss of muscle mass in sarcopenia is mainly characterized by type II muscle fiber atrophy, which is accompanied by a decrease in the amount of type II muscle fiber type-specific MuSCs in aging muscle (Leenders et al., 2013; Mackey et al., 2014; Verdijk et al., 2014). Based on studies showing that the onset of MuSC reduction precedes age-related muscle fiber atrophy (Brack et al., 2005) and MuSC content is a strong predictor of muscle fiber size in aged individuals (Verdijk et al., 2010), it has been hypothesized that age-related decreases in type II muscle fiber MuSC content may lead to decreased muscle fiber maintenance capacity, resulting in the specific type II muscle fiber atrophy observed in aged muscle (Snijders et al., 2009). Although it has been reported that muscle mass in humans is relatively stable between developmental maturity and 40 years of age, and MuSC function is often well preserved thereafter, signs of sarcopenia and a slow and steady decline in muscle mass can also occur (Mitchell et al., 2012). Compared with younger mice, old mice (20–24 months of age) showed a significant decrease in MuSCs along with fiber atrophy, but geriatric mice (28–32 months of age) showed no further loss of MuSCs when multiple sarcopenic symptoms increased (Sousa-Victor et al., 2014). Considering the conflicting evidence, it remains controversial whether the loss of MuSCs directly causes sarcopenia, although MuSCs are thought to be the sole source of new muscle nuclei and there is an inextricable link between the decay of MuSCs and poor muscle progression.

Fry et al. (2015) observed significant muscle mass loss only in flounder muscle after induced depletion of MuSCs from adult mouse skeletal muscle. In aged mice (24 months old), muscle atrophy consistent with criteria for human sarcopenia was observed in both control and MuSC-depleted groups, indicating that MuSC-dependent loss of regenerative capacity in adulthood does not accelerate sarcopenia in aging mice. However, there was a low frequency of MuSC depletion in this experiment (range 64%–87%) and normally aging control mice may still lack the muscle fiber regeneration that inhibits sarcopenia. Keefe et al. (2015) used a similar strategy to induce MuSC depletion in adult mouse skeletal muscle and found that MuSCs continued to contribute to muscle fibers even in the absence of injury, but were unnecessary to maintain muscle fiber CSA in uninjured adult muscle. The reduced number of muscle fibers in sarcopenia (Lushaj et al., 2008) is not mentioned in the MuSC-depleted mouse model.

The changes that muscles undergo over the lifetime of model mice cannot be fully analogous to humans, and a recent study based on human muscle samples is exciting. Although it is generally accepted that the decreased regenerative capacity of MuSCs correlates with aging (Forcina et al., 2019), Novak et al. (2021), who xenografted human muscle from different age sources into an immunodeficient mouse model, found that the muscle-forming capacity of MuSCs was not impaired with age and loss of muscle mass during aging was not attributable to any intrinsic myogenic loss of MuSCs. These findings confirmed that muscle regeneration can still occur even in MuSCs from elderly individuals in a friendly environment, suggesting the possibility of improving the environment for MuSC survival to protect muscle health.

Neuromuscular junctions (NMJs) are synapses located between motor neurons and skeletal muscle cells that are important for maintaining muscle mass and function (Liu et al., 2018). Degeneration of NMJs is associated with aging and one of the manifestations of sarcopenia. Depletion of MuSCs accelerates age-related degeneration of NMJs and significantly induces early onset of reduced cross-sectional area of muscle fibers, a sarcopenic phenotype (Liu et al., 2017). Activation of MuSCs in the vicinity of NMJs has been shown to be essential for promoting myofiber reinnervation and restoring muscle atrophy and fibrosis (Liu et al., 2015). Previous experiments demonstrated that overexpression of Spry1 in SCs attenuates age-related SC decline (Chakkalakal et al., 2012; Bigot et al., 2015). To verify that functional MuSCs are the key factor for maintaining muscle mass and strength, and slowing the progression of sarcopenia, Liu et al. (Liu et al., 2017) compared SC-specific Spry1-overexpressing Pax7 ^CreER/+^; CAG^Spry1/+^ (Spry1OX) mice with Pax7^+/+^; CAG^Spry1/+^ (control) mice. Flow cytometric analysis confirmed that aging muscles of Spry1OX mice had significantly more SCs at 12 months of age, and that Spry1OX muscles had diminished age-related neuromuscular junction degeneration and a robust increase in postsynaptic myonuclear cluster size. In behavioral tests, older Spry1OX mice had significantly longer run times and distances, and exhibited a significant increase in the peak absolute force produced by the muscle. This experiment demonstrates that retention of functional MuSCs in aging skeletal muscle is essential to maintain muscle mass and strength, and to slow the development of sarcopenia. Collectively, these findings lay the theoretical foundation for subsequent treatment of sarcopenia by improving MuSC function.

### 4.2 MuSC and muscle hypertrophy

As humans age, numbers of MuSCs decrease dramatically. Sarcopenia also elicits age-related loss of skeletal muscle, encompassing declines in both muscle fiber size and number. Experiments have validated the relationship between MuSCs and muscle hypertrophy, which is related to their potential to differentiate into muscle fibers.

In the 1990s, Rosenblatt and Parry used γ-irradiation to ablate MuSCs and observed virtually no muscle fiber growth in mice (Rosenblatt and Parry, 1992; Rosenblatt and Parry, 1993). However, due to controversy surrounding the low specificity of γ-irradiation on MuSCs, this test cannot fully prove a relationship between MuSCs and muscle hypertrophy. Therefore, in 2011, McCarthy et al. (2011) used the Pax7^CreER^-diphtheria toxin A (Pax7^CreER^-DTA) strain of mice that allows for conditional ablation of Pax7^+^ cells (MuSCs) to verify the hypothesis that MuSCs supports skeletal muscle hypertrophy. The results showed that muscle fiber hypertrophy induced by 2 weeks of overload was accompanied by the production of new myonuclei in muscle with MuSCs. However, similar hypertrophy of muscle fibers was observed in MuSC-ablated muscle. Therefore, the authors believe that MuSCs are not necessary for mouse skeletal muscle hypertrophy. In 2014, Fry et al. (2014) used the same mouse model and found that muscle fiber hypertrophy without satellite cells was impaired after 8 weeks of overload, providing a role for satellite cells in long-term muscle fiber hypertrophy. To explain the above controversy, Egner et al. (2016) used the exact same experimental model and found that increases of muscle fiber cross-sectional area and myonuclei contents were observed after 2-weeks overload, but not in MuSC-deficient muscles. The authors suggest that the reason for this discrepancy results from the use of different criteria for evaluating muscle fiber cross-sectional area in McCarthy’s experiment. McCarthy’s assessment included fibers showing signs of damage, whereas (Egner et al., 2016) observed that damaged muscle fibers were generally smaller than normal muscle fibers. If a large number of damaged muscle fibers are included in the statistics, it will mask the growth of muscle fibers in response to overload. Notably, (Egner et al., 2016) did observe a difference in muscle mass gain between normal and MuSC-ablated mice (112% and 55%, respectively), but they considered muscle mass to be an unreliable indicator of hypertrophy in the plantaris overload model due to complications after synergistic ablation. Hall et al. (2010) showed that transplantation of MuSCs (along with some muscle fibers) into injured muscles of young mice contributed to an increase in muscle mass and strength, and this positive effect persisted as the mice aged. In this transplantation model, the lifetime increases in muscle mass arose from an increase in muscle fiber number and progressive muscle fiber hypertrophy, providing new evidence that MuSCs can cause muscle hypertrophy.

Studies on the relationship between MuSCs and muscle hypertrophy in humans are mostly descriptive, however numerous studies have confirmed that the hypertrophy of muscle fibers during long-term resistance exercise is accompanied by increases in MuSCs and muscle nuclei content, as observed in both healthy young and old adults (Petrella et al., 2006; Leenders et al., 2013; Bellamy et al., 2014; Farup et al., 2014; Abou Sawan et al., 2021). Thus, MuSCs do indeed play an important role in muscle fiber hypertrophy under stimulation with human muscle growth factors, regardless of age.

These results demonstrate that MuSCs have a positive role in long-term muscle fiber hypertrophy, which is of great significance in resisting muscle loss associated with sarcopenia. Moreover, these findings suggest that optimizing the function of MuSCs with aging may be a feasible strategy to combat skeletal sarcopenia.

### 4.3 Interventions and mechanism of treating sarcopenia with MuSC

**FIGURE 4:**
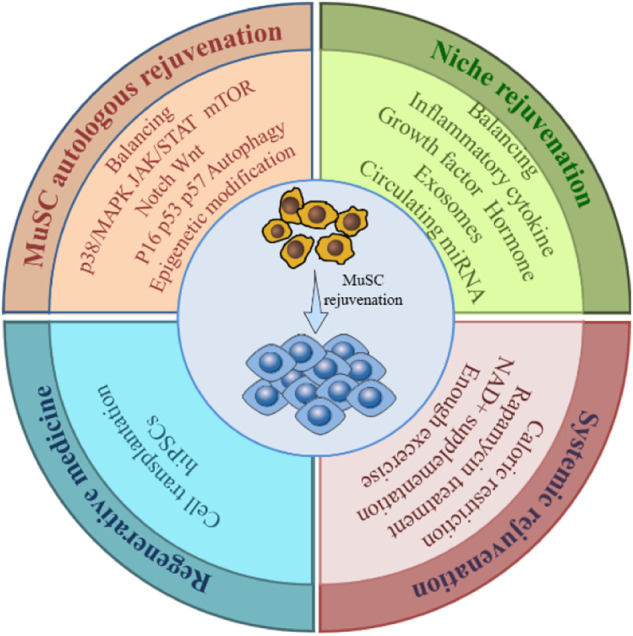
Rejuvenation strategies for MuSC. To restore the number and function of the aging MuSC requires restoring the overall rejuvenation of MuSC itself, niche and the system, by balancing dysregulated signaling pathways, cytokines, cellular metabolism, and genetic modifications etc., through various means. Cell transplantation and hiPSCs technologies in regenerative medicine also offer new approaches to MuSC rejuvenation.

The quantity and function of MuSCs are subject to a combination of intra- and extracellular signals. Therefore, restoration of MuSC function should be addressed from multiple perspectives (Figure 4).

#### 4.3.1 Strategies acting directly on MuSCs

Autonomous rejuvenation of MuSCs can be achieved by regulating cell cycle inhibitors, intracellular signaling pathways, autophagy, DNA damage, and epigenetics.

Expression of cell cycle inhibitors such as p16 ^Ink4^, p53, and p57 ^Kip2^ increases with aging, causing irreversible cell cycle arrest in MuSCs that severely affects their regeneration. Inhibition of p16 expression restores the asymmetric division potential of MuSCs and prevents their aging and depletion (Sousa-Victor et al., 2014). Given the ability of p53 to regulate the balance between quiescence, proliferation, and differentiation of MuSCs, inhibition of its expression could improve the proliferative capacity of MuSCs under certain diseases (Flamini et al., 2018; Kitajima et al., 2018).

As discussed above, p38/MAPK, JAK/STAT, Notch, mTOR, and other signaling pathways are aberrantly expressed within MuSCs during aging, and Stat3 has been identified to increase activity in sarcopenia (Tierney et al., 2015). Inhibition of p38/MAPK (Bernet et al., 2014) and JAK/STAT (Sorensen et al., 2018) signaling pathways, which are aberrantly activated in aging, rescues aging MuSC function. Conditional knockout of Stat3 in MuSCs confirms its critical role in regulating MuSC self-renewal during muscle regeneration (Zhu et al., 2016). Meanwhile, restoration of deficient Notch signaling could enhance MuSC stemness and proliferation (Conboy et al., 2005).

Autophagy plays an important role in cellular homeostasis by degrading intracellular waste through lysosomes. Autophagy provides temporary energy replenishment for activation and proliferation of MuSCs with low metabolic activity (Tang and Rando, 2014), whereas decreased autophagy levels due to senescence affect MuSC stemness. Balancing of autophagy and apoptosis in senescent MuSCs facilitates rescue of muscle regenerative capacity (White et al., 2018).

Histone H_2_A phosphorylation and comet tails, two DNA damage markers, were observed to become more abundant in MuSCs (Sinha et al., 2014; Liu et al., 2018). Accumulation of DNA damage in senescent MuSCs induces the onset of apoptosis, whereas activation of p53 inhibits the probability of DNA damage and apoptosis (Liu et al., 2018).

Although they do not alter the original gene sequence, epigenetic modifications control global gene expression patterns in cells and play a central role in cellular function. Young and aging MuSCs have different patterns of chromatin modifications (Liu et al., 2013), and these age-dependent epigenomic changes lead to decreased function of MuSCs. However, it has been demonstrated that histone deacetylase inhibitors can be used to treat stem cell senescence in MuSCs (Minetti et al., 2006).

#### 4.3.2 Strategies acting on the environment

The MuSC niche is the microenvironment with which they are in direct contact. Signal changes from the niche directly act on MuSCs to make them respond accordingly. Therefore, it is necessary to manage niche signals to reverse the aging of MuSCs and improve their function. The JAK/STAT pathway is involved in self-renewal of MuSCs (Sorensen et al., 2018), and studies have demonstrated that both Jak2 or Stat3 knockdown and pharmacological inhibition can significantly stimulate symmetric division of MuSCs on cultured muscle fibers (Price et al., 2014). Use of inhibitors such as sodium salicylate to target upregulation of the NF-κB signaling pathway with aging can inhibit chronic inflammation and improve the myogenic function of aging MuSCs (Oh et al., 2016). FGF2 induces QSCs to escape from their quiescent state, and inhibition of FGF2 by the FGFR1 inhibitor SU5402 or by increasing Spay1 expression helps rescue the senescence phenotype of MuSCs to improve their self-renewal ability (Chakkalakal et al., 2012). Injection of fibronectin, an extracellular matrix protein underexpressed in the senescent MuSC niche, can enhance the proliferative and myogenic potential of senescent MuSCs by activating the FAK signaling pathway (Lukjanenko et al., 2016). Interleukin-33 (IL-33) regulates muscle regulatory T (Treg) cells homeostasis in young mice. Muscle supplementation with IL-33 can improve defects in Treg cell recruitment and muscle regeneration following injury in elderly mice (Kuswanto et al., 2016). It should be noted that the presence of pro-inflammatory factors is essential for activation of MuSCs, and inflammatory pathways are an important regulator of muscle regeneration. For example, the inflammatory cytokine prostaglandin E2 (PGE2) can directly target MuSCs through EP4 receptors, resulting in MuSC proliferation. Inhibition of this PGE2 signal will damage muscle regeneration, and intramuscular injection of PGE2 can significantly enhance repair of damaged muscle (Ho et al., 2017). Therefore, we suggest that the above measures should be used with caution and moderation.

In addition to the direct effect of its resident niche, the function of MuSCs is also affected by cytokines, growth factors, hormones, exosomes, and circulating microRNA secreted by various cells in the circulation. Serum levels of TGF-β are elevated in aged mice and humans, leading to damage and aging of MuSCs. A TGF-β receptor kinase inhibitor restored the regeneration function of aging MuSCs by blocking TGF-β signaling (Carlson et al., 2009). Serum expression of Wnt ligands similarly exhibited an age-related rise. Activation of the Wnt signaling pathway mediated a change of lineage tendency during MuSC proliferation in elderly mice and antagonized Notch signaling. Intramuscular injection of a Wnt inhibitor can improve the proliferation potential of MuSCs (Brack et al., 2007). Expression of β1-integrin and fibronectin receptors on the surface of MuSCs is dysregulated in aged muscles (Brack et al., 2007). The function of aging MuSCs can be rescued by intramuscular injection of β1-integrin receptor activator or fibronectin (Lukjanenko et al., 2016; Rozo et al., 2016). Granulocyte colony-stimulating factor (G-CSF) receptors are asymmetrically expressed in activated MuSCs. Experiments confirm that G-CSF is essential for long-term muscle regeneration and functional maintenance in mouse models of Duchenne muscular dystrophy (Hayashiji et al., 2015). WNT1 inducible signaling pathway protein 1 (WISP1) controls the expansion and asymmetric commitment of MuSCs through Akt signaling, which is essential for effective muscle regeneration and lost during aging. Systemic treatment with WISP1 can restore the myogenic capacity of MuSCs and rescue skeletal muscle regeneration in aged mice (Lukjanenko et al., 2019). Oxytocin levels in plasma decrease with age. Genetic deficiency of oxytocin will lead to the premature occurrence of sarcopenia. Inhibition of oxytocin signaling in young mice reduces muscle regeneration, whereas systemic administration of oxytocin improves muscle regeneration by activating the MAPK/ERK signaling pathway and enhancing proliferation of aging MuSCs (Elabd et al., 2014). Epigenetic control of the “anti-aging” protein α-Klotho promoter is lost with age. Exogenous supplementation of α-Klotho restores MuSC mitochondrial DNA integrity and energy supply to enhance muscle regeneration (Sahu et al., 2018).

Aging is a process of progressive loss of physiological integrity over time that manifests at multiple levels, including genomic instability, telomere shortening, loss of heterochromatin, programmed aging, and free radical accumulation. Interventions targeting rejuvenation of the whole living organism will likewise have a positive impact on the abrogation of MuSC function due to aging. Caloric restriction has been shown to extend lifespan and ameliorate age-related diseases in most species, and can have beneficial effects on mitochondrial function and apoptotic signaling in skeletal muscle of both young and old animals (Cerletti et al., 2012). Statistics show that even a reduction in food intake of as little as 8% still provides protection against sarcopenia (Marzetti et al., 2008), and both short- and long-term caloric restriction enhances myogenic cell proliferation (Abreu et al., 2020). Activation of mTOR signaling is one of the mechanisms of aging and many studies have confirmed that rapamycin can improve aging-related diseases and effectively prolong lifespan by inhibiting the mTOR pathway. Evidence suggests that rapamycin treatment restores age-related muscle fiber loss (Tang et al., 2019) and rescues exercise capacity (Fischer et al., 2015; Xue et al., 2016) by inhibiting the AKT-mTORC1 signaling pathway. Increasing circulating NAD^+^ levels through supplementation with NAD^+^ precursors such as nicotinamide riboside has been shown to have a delaying effect on aging (Zhang et al., 2016). Recent experiments showed that the NAD^+^-dependent deacetylase SIRT2 mediates deacetylation of PAX7, leading to an increase in asymmetric cell division and stabilization of the MuSC pool, thus contributing to the maintenance of skeletal muscle homeostasis (Sincennes et al., 2021).

Finally, it is important to mention exercise, a measure that has been shown to improve overall health and promote MuSC function in multiple ways. Apelin induced by muscle contraction during exercise has been found to promote MuSC mediated muscle repair (Vinel et al., 2018). In fact, resistance and endurance training have different degrees of MuSC mobilization function (Ingjer, 1979; Mackey et al., 2009). However, the mechanism by which exercise activates MuSCs remains largely unknown, but it may reshape the MuSC niche by inducing local micro-injuries and the release of inflammatory cytokines and growth factors to maintain MuSC activity, thus maintaining skeletal muscle health.

#### 4.3.3 Strategies of regenerative therapies

Cell transplantation therapy is one strategy to maintain a functional MuSC pool. First, there is evidence that direct transplantation of highly purified muscle stem cells can ameliorate muscle degenerative disease. A subpopulation of CSM4B sorted on the basis of cellular muscle satellite cell surface markers is capable of stable clonal myogenic differentiation in cell culture assays. Direct isolation and transplantation of this subpopulation into mdx mice (a model of Duchenne Muscular Dystrophy) and cardiotoxin-injured wild-type mice not only reconstituted mature muscle fibers, it replenished the primitive muscle precursor pool for future muscle growth and repair (Cerletti et al., 2008). Human induced pluripotent stem cells (hiPSCs) can be induced to differentiate into myogenic cells and mature skeletal muscle cells (Kodaka et al., 2017), thus offering a good solution to problems associated with transplanting cells from other donor sources, including ethics and immune rejection. In addition, the patient’s original genetic mutations can be corrected in hiPSCs by gene editing technology (van der Wal et al., 2018), so hiPSCs have great potential for clinical applications. Generation of human myogenic cells from hiPSCs can be achieved by overexpression of myogenic transcription factors (e.g., PAX3 or PAX7, and MyoD) by means of lentiviral vectors to mimic the induction pattern of mesoderm and activate myogenic cells (Roca et al., 2015). This technique allows for large-scale generation of human myogenic cells, but has the pitfall of integration of lentiviral gene sequences in the host genome, which limits clinical translation. Use of small molecule induction protocols such as WNT, FGF, and BMP avoids the introduction of viral vectors, but is inefficient and difficult to control the purity of the induced cell population (Wang et al., 2019). In the last two years, studies have pointed to the genomic safe harbor expression of PAX7 for induction of myogenic programs in hiPSCs to generate transplantable myogenic cells, providing a new idea for clinical application of hiPSCs (Kim et al., 2021).

## 5 Conclusion and perspective

The etiology of sarcopenia is diverse and has a significant impact on the quality of life of older people. Current treatments for sarcopenia focus on physical exercise, nutritional supplementation and pharmacological interventions, but these approaches are not suitable for everyone and do not completely curb sarcopenia. Therefore, it is necessary to find new treatments for sarcopenia. Although it has been more than 70 years since the discovery of MuSC, the complex and delicate regulation of MuSC and its enormous potential applications is still fascinating. Numerous animal experiments as well as human-based cross-sectional studies have described the relationship between sarcopenia and MuSC, suggesting the possibility of treating sarcopenia by improving MuSC. However, we are still on the verge of deciphering the regulation of MuSC activation, proliferation and differentiation during muscle degeneration during aging. Many questions remain: What are the similarities and differences between the MuSC that can be captured by current experimental methods and its state *in vivo*? What changes and roles do MuSC subpopulations undergo during muscle aging? Can it use as a target for future therapies? With the application of new technologies such as bioinformatics, *in situ* sequencing and 3D imaging, these questions will be answered perfectly in the future. Since many questions are still unclear, we propose an improvement scheme for endogenous repair of aging MuSC combined with rejuvenation of niche signaling as well as circulatory signaling in the organism to act integrally to prevent, delay or reverse the loss and dysfunction of MuSC in relation to sarcopenia, thus achieving a long and healthy life in older people.
